# RF Magnetron Sputtering of Substituted Hydroxyapatite for Deposition of Biocoatings

**DOI:** 10.3390/ma15196828

**Published:** 2022-10-01

**Authors:** Konstantin A. Prosolov, Vladimir V. Lastovka, Margarita A. Khimich, Valentina V. Chebodaeva, Igor A. Khlusov, Yurii P. Sharkeev

**Affiliations:** 1 Laboratory of Physics of Nanostructured Biocomposites, Institute of Strength Physics and Materials Science, Siberian Branch of Russian Academy of Sciences, 634055 Tomsk, Russia; 2 Laboratory of Cellular and Microfluidic Technologies, Siberian State Medical University, 634050 Tomsk, Russia; 3 Research School of High-Energy Physics, National Research Tomsk Polytechnic University, Lenin Avenue 30, 634050 Tomsk, Russia

**Keywords:** antibacterial effect, biocompatibility, calcium phosphate, ion substitution, physical vapor deposition, thin films

## Abstract

Functionalization of titanium (Ti)-based alloy implant surfaces by deposition of calcium phosphates (CaP) has been widely recognized. Substituted hydroxyapatites (HA) allow the coating properties to be tailored based on the use of different Ca substitutes. The formation of antibacterial CaP coatings with the incorporation of Zn or Cu by an RF magnetron sputtering is proposed. The influence of RF magnetron targets elemental composition and structure in the case of Zn-HA and Cu-HA, and the influence of substrate’s grain size, the substrate’s temperature during the deposition, and post-deposition heat treatment (HT) on the resulting coatings are represented. Sintering the targets at 1150 °C resulted in a noticeable structural change with an increase in cell volume and lattice parameters for substituted HA. The deposition rate of Cu-HA and Zn-HA was notably higher compared to stochiometric HA (10.5 and 10) nm/min vs. 9 ± 0.5 nm/min, respectively. At the substrate temperature below 100 °C, all deposited coatings were found to be amorphous with an atomic short-range order corresponding to the {300} plane of crystalline HA. All deposited coatings were found to be hyper-stochiometric with Ca/P ratios varying from 1.9 to 2.5. An increase in the substrate temperature to 200 °C resulted in the formation of equiaxed grain structure on both coarse-grained (CG) and nanostructured (NS) Ti. The use of NS Ti notably increased the scratch resistance of the deposited coatings from18 ± 1 N to 22 ± 2 N. Influence of HT in air or Ar atmosphere is also discussed. Thus, the deposition of Zn- or Cu-containing CaP is a complex process that could be fine-tuned using the obtained research results.

## 1. Introduction

New materials for regenerative medicine are in high demand due to the aging of the ever-increasing population [1]. The most important parameter among others for newly developed materials ready to be introduced into clinical practice is their biocompatibility [2]. Biocompatible materials can be divided into three groups: biotolerant, bioinert, and bioactive [3]. Bioinert alloys, a striking representative of which are titanium (Ti)-based alloys, are characterized by osteoconductive property, which means that these materials are not included into the metabolic process, are not dissolved in the body, but their surface can provide a physical and mechanical connection to body tissues, and their oxide film promotes the adhesion of various proteins that trigger the osseointegration process [4]. On the other hand, it is known that the wear of the metal surface of implants occurs during their service life, e.g., the cobalt-chrome-molybdenum alloy wears at an average rate of 0.02–0.06 mm over 10 years. The metal chips or particles that appear during wear are phagocytized by macrophages, which produce IL-1, IL-6, TNF, PGE2, and other cytokines, causing a cascade of immune reactions [5]. The same goes for Ti-based alloys, in the work by Kovac et al. [6], where metal ions release after orthodontic appliances were studied, a risk of the local inflammatory process was confirmed. At present, one of the main tasks of medical materials science is to study the wear mechanisms and minimize the formation of metal particles during the implant lifetime [7], including the application of protective coatings.

**Figure 1:**
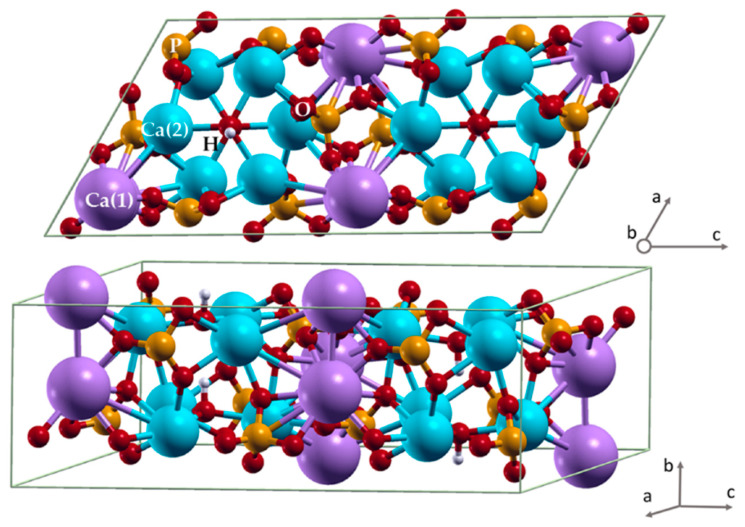
Hydroxyapatite unit cell. Color coding: phosphorus—orange, oxygen—red, hydrogen—light grey, Ca(1)—purple, Ca(2)—blue [11].

In this regard, the deposition of calcium phosphates (CaP) can prevent implant corrosion and the formation of wear debris while simultaneously providing the desired bioactivity. Such bioactive materials promote bone tissue regeneration and, due to the metabolism of the bone matrix, upon dissolution are partially or completely replaced by bone tissue over time. Various CaP are striking examples of the bioactive materials group [8]. One of the properties of CaP, and especially hydroxyapatite (HA, Ca_10_(PO_4_)_6_(OH)_2_), is the ability of ion substitution in the anionic and cationic sublattices of HA and the occurring variability of structural characteristics and physicochemical properties of this material. Even though HA is the most widely used material among all other CaP materials in clinical practice [9], a more promising approach is to use the substituted HA which could be fine-tuned for a specific clinical case due to the properties of the substituent ion [8]. Substitutions in the apatite structure have been developed for a wide range of biomedical applications, such as bone repair and tissue regenerations; bioactive and/or antibacterial coating for medical devices, biomarkers, or carriers in drug/gene delivery systems; and biomagnetic agents for cancer treatment [10]. These properties are due to the unique structural characteristics of HA, the unit cell of which is represented in Figure 1 [11]. HA belongs to the class of minerals whose composition has a general formula: M_10_(ZO_4_)_6_X_2_, where M is one-, two-, three-valent cations (K^+^, Ca^2+^, Sr^2+^, Ba^2+^, Pb^2+^, Na^+^, Mn^2+^, Mg^2+^, Th^3+^, Ni^2+^, etc.); ZO_4_ is one-, two-, three-valent cation. ); ZO_4_—mono-, bi- and trivalent anions (PO_4_^3−^, SiO_4_^3−^, CO_3_^2−^, AsO_4_^3−^, SO_4_^2−^,VO_4_^3−^ etc.); X—mono- and divalent anions (F^−^, Cl^−^, OH^−^, O^2−^, CO_3_^2−^ etc.) [12].

Currently, a large number of scientific groups is engaged in the manufacturing and application of HA-based bio-coatings with partial substitution of Ca^2+^ cations for other metal cations, such as Ag^2+^, Si^2+^, Sr^2+^, Cu^2+^, and Zn^2+^ in the structure, since such substitutions significantly change the physicochemical and biological properties of HA. However, Zn^2+^ and Cu^2+^ substitutions appear to be some of the most promising approaches for overcoming the ever-existing problem of septic instability of implants and various infections [8]. It is established that these ions contribute to the processes of osteogenesis (bone formation) [13]. Zn^2+^ promotes bone formation and regeneration by supporting the activity of osteoclasts and reducing the activity of osteoblasts [14]. The influence of Cu^2+^ is characterized by substantial biological activity [15]. This cation is also antibacterial and promotes osteogenesis. However, it has been reported that Cu^2+^ is a more “active” cation when compared to Zn^2+^ and has a higher potential for cytotoxicity at relatively high concentrations [16]. Cu-substituted HA (Cu-HA) was found to have good barrier characteristics and provide corrosion protection for Ti substrates [17]. While it is possible to manufacture Zn-substituted HA (Zn-HA) or Cu-HA by a “wet”-chemical method, it is also possible to obtain it by solid-phase synthesis (mechanochemical synthesis), as described in [15]. For example, it has been established that by using the Zn(H_2_PO_4_)_2_ 2H_2_O reactant during the synthesis, it is possible to substitute up to x ≤ 0.8 in the Ca_10-x_Zn_x_(PO_4_)_6_(OH)_2_ structure without the production of secondary phases, namely amorphous calcium phosphate (ACP) or tricalcium phosphate (TCP, Ca_3_(PO_4_)_2_). Substituted HA having Cu^+^ and Cu^2+^ in the lattice could also be produced by mechanochemical synthesis [18]. Thus, in the present study, we focused on the manufacturing of Zn-HA and Cu-HA using mechanochemical synthesis for subsequent use in coatings deposition.

There are a number of methods of CaP coating deposition. These methods include plasma spraying [19], micro-arc oxidation or plasma electrolytic oxidation (PEO) [20,21], laser ablation [22], and RF magnetron sputtering [23,24]. All of the above methods have their advantages and disadvantages, but it is worth noting that today, more and more research is focused on the formation of coatings by physical vapor deposition (PVD) methods. An RF magnetron sputtering, a striking example of the PVD family of methods for CaP deposition allows to greatly vary the functional properties of the coatings, precisely control the chemical composition, and obtain coatings with a high degree of adhesion to the substrate, which is certainly a key factor for their application in medicine. In a recent report by Kozelskaya et al. [25], an RF magnetron sputtering of Mg- or Sr-substituted HA and TCP targets for the functionalization of CaP PEO coatings resulted in multilevel roughness. However, the structure of sintered targets and the deposition rate are not reported. Similarly, a deposition rate in relation to stochiometric Zn-HA is not reported in the work following the report [26], where the initial powder was obtained by wet chemical precipitation. Even at a relatively high 10 mol.% of substitution in HA lattice, sintering of the target did not result in the formation of byproducts or secondary phases. However, the exact position of Zn ions in the HA lattice is hard to confirm. Contrary to that, sintering of the Zn-HA powder, also obtained by wet chemical precipitation, with a Zn concentration of 7.8 at.% at 1150 °C resulted in the formation of multiphased target comprised of ZnO, CaO, TCP, and HA phases as reported in [27]. Hence, the target sintering conditions, the method of substituted HA manufacturing and substitution concentrations significantly affect the resulting target material. Hence, a more thorough study in this regard is needed. In our work, mechanochemical synthesis for obtaining HA, Zn-HA, and Cu-HA and subsequent target formation was used.

Moreover, it is still debated which state of bioactive coatings should be used in clinical practice: crystalline, amorphous, or nano-bio-composite, which is a mixture of both phases. It has been demonstrated that the rate of HA osteointegration with bone depends not only on the composition, but also on the rate of calcium and phosphate ions released from the HA structure, which is the determining factor in the establishment of a strong implant-bone integration [28]. It has been demonstrated in a number of studies that the dissolution rate of the coatings decreases with a decrease in the fraction of ACP. If there is a need for rapid release of bioavailable calcium phosphate, it is advisable to use ACP-based coatings. However, in [29], it was shown that ACP causes high monocyte adhesion and increased TNF-α secretion indicates increased undesirable local inflammatory activity. In this regard, the use of ACP in vitro is not always accompanied by a positive result. On the other hand, a recent in vivo study showed that Ti-based implants with controlled nanotopography (hemispherical structural elements not exceeding 80 nm in size) result in less macrophage adhesion and low local inflammatory activity, which suggests that ACP is promising for in vivo rather than in vitro conditions. Indeed, the high dissolution rate does not always have a negative effect on bio-objects. Thin ACP coatings have been shown to significantly enhance osteogenesis compared to crystalline HA. This effect is due to the rapid dissolution of ACP and the release of ions that are both therapeutic agents and building blocks of new bone tissue [30]. It was noticed that the topography and chemical composition of the surface of amorphous ACP and substituted HA coatings significantly increase cell adhesion without disturbing the vital activity of cells [31]. It was found [32] that ACP deposited on the implant surface can be beneficial for local physiological activity and can stimulate active bone growth. It is reported that the dissolution of ACP is a necessary preliminary stage of bone tissue transformation and healing. In [33], it was found that the deposition of biological HAs in vivo is observed only in the case of the HA coating with a low degree of crystallinity. On the other hand, it is noted that HA coatings with a high degree of crystallinity have the most favorable effect on osteoblast proliferation, since they are almost insoluble in the culture medium and, therefore, represent a stable surface to which cells can adhere under in vitro conditions. However, such experiments usually do not take into account the influence of osteoclasts, cells that remove bone tissue through a dissolution of its mineral component and hence the dissolution of CaP coatings. It is known that experiments performed in vitro are not always successfully translated and have predictable results under in vivo conditions [34]. For example, according to recent data, no statistically accurate difference between the success rate of CaP coatings with variable degrees of crystallinity (55% vs. 98%) obtained by plasma spraying has been established [35]. The work also noted the risk of forming relatively thick (100 μm) amorphous-crystalline coatings. The risk is related to the fact that while the amorphous phase dissolves, the crystalline phase in the form of particles is released from the coating volume and accumulates in the body tissues, which can cause an undesirable immune reaction. Some published papers discuss computational studies regarding implant wear [4], the formation of CaP in the liquid phase [36], and the structure and properties of defected HA [37]. Thus, it is important to be able to fine-tune the formation of CaP coatings in terms of their crystallinity and elemental composition for further application in specific clinical cases with the aid of computational studies.

In the available literature, however, the structural characteristics of sintered targets from substituted HA and their influence on the deposition rate are rarely discussed [25,26,27]. Even though the deposition rate is crucial for tailoring the needed properties of deposited layers. Little attention is paid to the thin structure of deposited coatings, the predominant majority of papers related to CaP deposition analyze its structure using X-ray diffraction (XRD) or Fourier transform infrared (FTIR) analysis while transmission electron microscopy (TEM) studies, which could be found elsewhere [24,27,38,39], are very limited, although it could provide more insightful information. Post-deposition HT is routinely used for the improvement of crystallinity [24,24,40]. However, a comparison of structure evolution after HT in different ambient conditions such as air or Ar gas is rarely discussed. Lastly, nanostructured Ti is becoming more widely used in clinics due to its high mechanical properties [41,42]; however, the influence of substrates’ crystallinity state on the mechanical properties and crystallinity of deposited coatings is rarely discussed.

In our study, we tried to explore the physicomechanical properties of CaP coatings after sputtering Zn-HA and Cu-HA at the highest possible concentrations that mechanochemical synthesis could provide minding the fact that the obtained targets should contain only a single phase of HA even after sintering. In the present paper, we report the research results of a comprehensive study that describes the influence of targets’ elemental and phase composition in the case of Zn-HA and Cu-HA, the structural state of Ti substrates, gas atmosphere during post-deposition heat treatment, and in situ heating on Zn or Cu containing CaP coating’s structure and properties.

## 2. Materials and Methods

**Figure 2:**
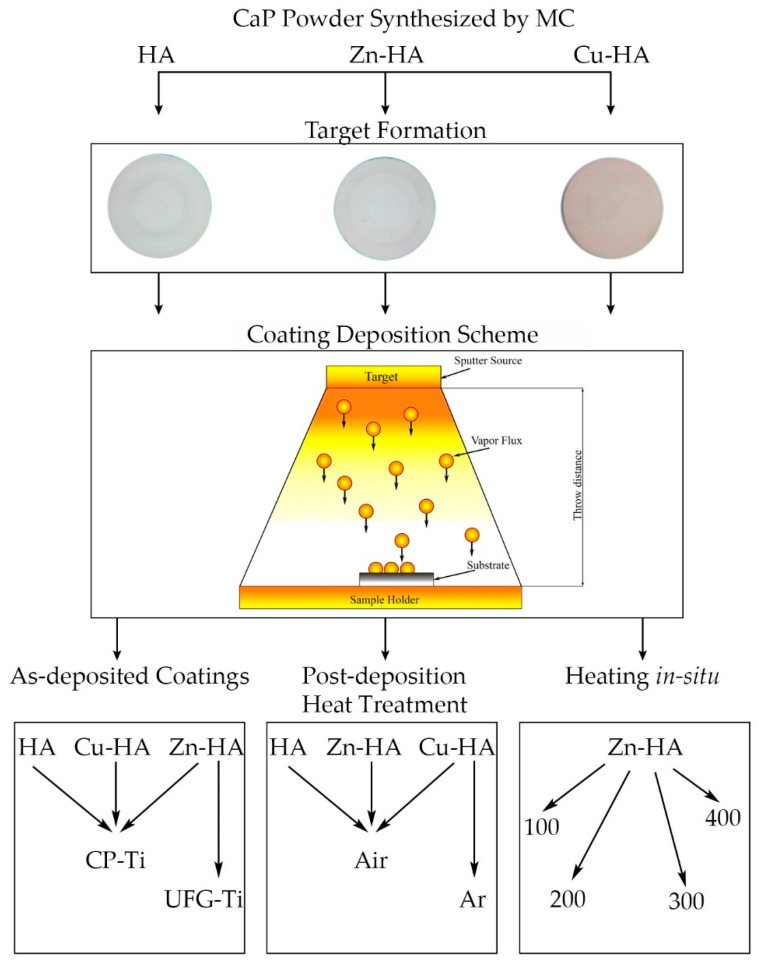
Workflow of the study.

The workflow of the current study is schematically represented in Figure 2. A detailed description of each of these stages is given below in Section 2.1 and Section 2.2.

### 2.1. RF Magnetron Target Manufacturing

Targets for magnetron sputtering were fabricated from HA powders (Ca_10_(PO_4_)_6_(OH)_2_), Cu-HA (Ca_9.8_Cu_0.2_(PO_4_)_6_(OH)_2_), and Zn-HA (Ca_9.6_Zn_0.4_(PO_4_)_6_(OH)_2_). 

The starting materials for targets based on Zn-HA and Cu-HA were powders obtained by mechanochemical synthesis (MC) in the Laboratory of Intercalation and Mechanochemical Reactions of the Institute of Solid State Chemistry and Mechanochemistry of the Siberian Branch of the RAS, Novosibirsk [43]. In mechanochemical synthesis, chemical reactions are initiated by the energy released during the collision of balls in a special planetary ball mill under the action of friction forces. To obtain powders of stoichiometric HA and HA with substitutions, a planetary ball mill with three steel drums, each with a volume of 2000 mL, was used. The duration of the process was 25–30 min. The reactions by which the synthesis took place are presented below:

$$6{\mathrm{CaHPO}}_{4}+4\mathrm{CaO}={\;\mathrm{Ca}}_{10}{\left({\mathrm{PO}}_{4}\right)}_{6}{\left(\mathrm{OH}\right)}_{2}\cdot 2H_{2}O;$$

$$6{\mathrm{CaHPO}}_{4}+3.8\mathrm{CaO}+0.2\mathrm{CuO}={\mathrm{Ca}}_{9.8}{\mathrm{Cu}}_{0.2}{\left({\mathrm{PO}}_{4}\right)}_{6}{\left(\mathrm{OH}\right)}_{2}\cdot 2H_{2}O$$

$$5.2{\mathrm{CaHPO}}_{4}+4.4\mathrm{CaO}+0.4\left(\mathrm{Zn}{\left(H_{2}{\mathrm{PO}}_{4}\right)}_{2}\cdot 2H_{2}O\right)={\mathrm{Ca}}_{9.6}{\mathrm{Zn}}_{0.4}{\left({\mathrm{PO}}_{4}\right)}_{6}{\left(\mathrm{OH}\right)}_{2}\cdot 3.2H_{2}O$$

The targets from the obtained powders were formed by uniaxial pressing in steel molds using a MIS-6000.4K hydraulic press (OOO IMASH, Armavir, Russia). A four-stage pressing mode was chosen with maximum pressure in the mold at the last stage of 65–75 MPa. Sintering of the targets was conducted in an air atmosphere in an ITM 12.1200 electric furnace (OOO ITM, Tomsk, Russia). When choosing the modes of sintering of pressed targets, we used the recommendations on the regularities of sintering given in the review by S.M. Barinov and V.S. Komlev [44], and the results of earlier dilatometric studies of shrinkage during annealing. The maximum sintering temperature was 1150 °C, the heating cycle to the maximum temperature was 4 h, and the cooling cycle was more than 30 h. The resulting ceramic targets made of HA, Cu-HA, and Zn-HA have a diameter of 110 mm. After sintering targets were mounted on a magnetron sputtering device.

### 2.2. RF Magnetron Deposition on Titanium Substrates

The substrates for deposition in the present work were as received titanium grade 2 (Ti) produced in Russia (VSMPO-AVISMA, Verkhnyaya Salda, Russia) both in the coarse-grained (CG) and nanostructured state (NS Ti) according to a protocol of severe plastic deformation reported elsewhere [45], with dimensions of 10 mm × 10 mm × 1 mm.

Before the stage of coating deposition by the RF magnetron sputtering method, all samples were mechanically processed using grinding papers of the following grades P400, P600, P1000 (GOST 6456-82). Next, the samples were polished using diamond pastes with an abrasive capacity of 14/10 and 5/3 (GOST 25593-83). After polishing, the surfaces of Ti disks were subjected to ultrasonication in acetone, soapy water, and alcohol. After that, the samples were dried at room temperature. During each deposition process, the silicon wafer (100) (Si) 10 mm × 10 mm in size was also deposited for further ellipsometry studies.

In this work, an RF (13.56 MHz) magnetron installation was used for the formation of CaP coatings. The unit is equipped with a 2.5 kW RF power supply (COMDEL CX-2500S, Gloucester, MA, USA with an automatic matching network (COMDEL Match pro CPMX-2500, Gloucester, MA, USA). CaP coatings were deposited in a vacuum chamber where the pressure of working gas Ar was controlled by a gas flow meter and was set to 20 sccm which resulted in 0.1 Pa during deposition. The geometry of the location of the magnetrons relative to the substrate holder makes it possible to deposit coatings at various distances from the target surface to the samples in the range from 40 to 120 mm. It also resulted in different heating of the substrates during deposition. At the distance of 40 mm the substrate temperature reached 200 °C without an additional source of heating according to chromel-alumel thermocouple, while at the throw distance of 60 mm and more, the substrate temperature was not higher than 100 °C. The substrate holder was under a floating potential and in the process of deposition of coatings on the samples is located under the magnetron target. The samples were deposited in stationary mode, i.e., no rotation of the substrate holder was performed. The duration of the deposition process varied in the range from 120 to 240 min, and the supplied RF power from 150 to 350 W in order to study the kinetics of coatings growth. Before each deposition run, the target in use was pre-sputtered by gradually ramping up the supplied RF power with a rate of 50 W per 5 min.

To study the effect of post-deposition HT on the structure and properties of CaP coatings, a furnace with a quartz tube was used. The coatings were subjected to HT both in air and in a protective Ar gas atmosphere. In the case of HT in a protective Ar atmosphere, the samples with RF coatings were placed in a quartz tube. The volume of the tube was evacuated to a residual pressure of 1 Pa using a rotary pump, followed by the protective Ar gas to a pressure of 35 kPa. The duration of isothermal exposure at temperatures of 400 °C and 700 °C was 3 h in a protective Ar atmosphere. The duration of isothermal holding in air at a temperature of 700 °C was 1 h. The rate of heating and cooling did not exceed 15 °C/min.

### 2.3. Materials Characterization

To study the structure of the obtained HA powders, targets after sintering, and resulting RF magnetron coating, an X-ray diffraction (XRD) method was chosen. To determine the structural-phase state of powder material and sputtering targets, we used a DRON-7 diffractometer (Bourevestnik, St. Petersburg, Russia) in the range of angles 2θ = 10–100° with scanning step 0.02° in Co-Kα radiation at accelerating voltage 35 kV and cathode current 22 mA. Step accuracy of DRON-7 is 0.0005°. To determine the lattice parameters, the size of coherent scattering regions (CSRs), and the magnitude of internal stresses, the full-profile analysis Rietveld method was used. In all the cases, R_exp_^2^-factor was not less than 0.96. Calculations of the lattice parameters and phase relations were performed using the program of full-profile analysis Powder Cell 2.4 (Federal Institute for Materials Research and Testing, Berlin, Germany). XRD study was performed in standard Bregg–Brentano geometry. Standard ICDD PDF 4+ (International Centre for Diffraction Data, Newtown Square, PA, USA) cards were used for phase analysis: HA (00-009-0432), Ca_3_(PO_4_)_2_ (00-032-0176), and Ti (00-044-1294).

To study an RF magnetron-deposited coating an X-ray diffractometer Shimadzu XRD 6000 (Shimadzu, Kyoto, Japan) with Cu-Kα-radiation in the range of angles 2θ = 5–90° with scanning step 0.04° at accelerating voltage 40 kV and cathode current 30 mA was used. Shimadzu step accuracy is 0.0001°. Cu-radiation was applied for studies of deposited coatings, as it allows for reducing the background in XRD patterns and reveals more phases due to the deeper penetration of the X-ray beam into the sample. The degree of crystallinity of the coatings was evaluated using the method described in [46,47]. The crystallinity index of CaP coatings was calculated using the following formula:

(1) $$Crystallinity\;index=\frac{I_{coating}}{I_{target}}*100\%,$$

where *I_coating_* is the sum of intensities of the main HA reflexes determined on the X-ray profile of the coatings, namely (002), (210), (211), (112), (300), (202) planes, and *I_target_* is the sum of intensities of the corresponding reflexes from the target. In this method, the degree of crystallinity of the target is considered to be equal to 100%.

The coatings’ thickness was determined by ellipsometry on Si samples made of monocrystalline polished silicon with dimensions of at least 5 mm × 5 mm. The measurements were carried out on a spectral ellipsometric complex ELLIPS-1891 SAG (SPC “Nanotechnology center”, Novosibirsk, Russia). At least six samples related to six deposition runs per sputtering target working at the same sputtering conditions were evaluated and results are presented as mean ± SD. To study the adhesion of CaP coatings, a benchtop measuring scratch macro tester Revetest RST (CSM Instruments, Needham Heights, MA, USA) was used. In each measurement, the scratching track length was 7 mm, and the load on the indenter with a radius of curvature of 20 μm varied from 10 to 30 N. At least three measurements per sample were performed. The results are reported as mean ± SD. A Zeiss Libra 200 Transmission Electron Microscope (Zeiss, Jena, Germany) and a JEOL JEM-2100 (JEOL, Akishima, Japan) transmission electron microscope were used to study the microstructure of the CaP coatings. The guaranteed resolution of Libra 200 is 0.12 nm and of JEM2100 is 0.14 nm. Preparation of the samples for TEM was performed by ion thinning at low angles and low energies, to minimize the effect on the structure of the coatings, on the Jeol Ion Slicer EM-09100 IS (JEOL, Akishima, Japan).

## 3. Results

### 3.1. Targets for RF Magnetron Sputtering

**Figure 3:**
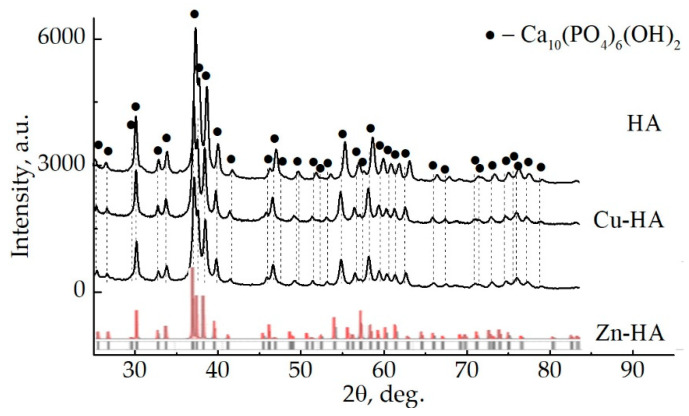
XRD patterns of HA, Cu-HA, and Zn-HA powders after mechanochemical synthesis (red and gray lines represent HA etalon XRD-pattern according to PDF-card no. 00-009-0432).

Before the target synthesis step, the initial Zn-HA, Cu-HA, and HA powders after mechanochemical synthesis were analyzed. XRD of Zn-HA and Cu-HA powders compared to stoichiometric HA are shown in Figure 3. The XRD pattern contains reflections of the crystalline phase of HA, without residual phases or byproducts of synthesis. The HA peaks, according to the ICDD database are shown by dotted lines. As can be seen, the positions of some peaks are shifted, which may indicate changes in the lattice parameters of the HA phase in all powders under study. At the same time, a significant broadening of the peaks and redistribution of intensities are noticeable.

The process of mechanochemical synthesis is a high-energy mechanical processing of powder material, during which, in addition to mixing, particle deformation occurs, which leads to an increase in internal stresses in the processed powder, as well as to significant refinement of its structure and an increase in the amount of defect. Broadened peaks with reduced intensity compared to the reference ICDD profile may indicate high internal stresses and small values of coherent scattering regions (CSR) in the resulting powders. It is known from the literature that HA has a hexagonal crystal lattice [48]. According to [12], the lattice parameters of pure HA are: *a* = *b* = 9.422 Å, *c* = 6.881 Å, *c*/*a* = 7.158, V = 533.7 Å^3^. The incorporation of Cu^2+^ or Zn^2+^ ions occurs by the mechanism of isomorphic substitution with charge conservation. For example, substituent cations Cu^2+^ or Zn^2+^, as a rule, replace Ca^2+^ in position (II) of the HA crystal lattice [12]. It is worth noting the difference in ionic radii of ions, as the average ionic radius of Ca^2+^ is 0.100 ± 0.003 nm, while the ionic radius of Cu^2+^ and Zn^2+^ is 0.075 ± 0.002 nm and 0.076 ± 0.002 nm, respectively [49]. It should be expected that a significant difference in ionic radii will affect the unit cell volume of substituted HA. To determine the change in the unit cell parameters in the case of substituted Cu-HA and Zn-HA, a full profile analysis was conducted. The results of calculating the parameters of the structure of powders after mechanochemical synthesis are presented in Table 1.

As can be seen from the results presented in Table 1, the lattice parameter *c* for substituted HA is significantly smaller than for stoichiometric HA. Many researchers have noted a trend towards an increase in the lattice parameter *a* with a simultaneous decrease in the lattice parameter *c* in cases of substitution of Zn in the composition of the HA structure up to 5 mol.% [13,50]. This is also confirmed by our research. Along with this, the unit cell volume for Cu-HA and Zn-HA is 519.9 Å^3^ and 513.4 Å^3^, respectively, which is significantly less than the cell volume of stoichiometric HA obtained in the course of mechanochemical synthesis (528.1 Å^3^). The volume of the unit cell naturally decreases due to the significant difference in the ionic radii of the Ca^2+^, Cu^2+^, and Zn^2+^ cations. Since after the synthesis of Cu-HA and Zn-HA, byproducts of the reaction (CuO or ZnO) are not detected, and the parameters of the crystal lattice of HA change significantly, it can be concluded that an isomorphic substitution of the Ca^2+^ ion in position (II) on Cu^2+^ or Zn^2+^ in the crystal lattice of HA occurred. At the same time, Cu^2+^ or Zn^2+^ ions inhibit the growth of HA crystallites, which can be seen from the decrease in the CSR sizes.

**Figure 4:**
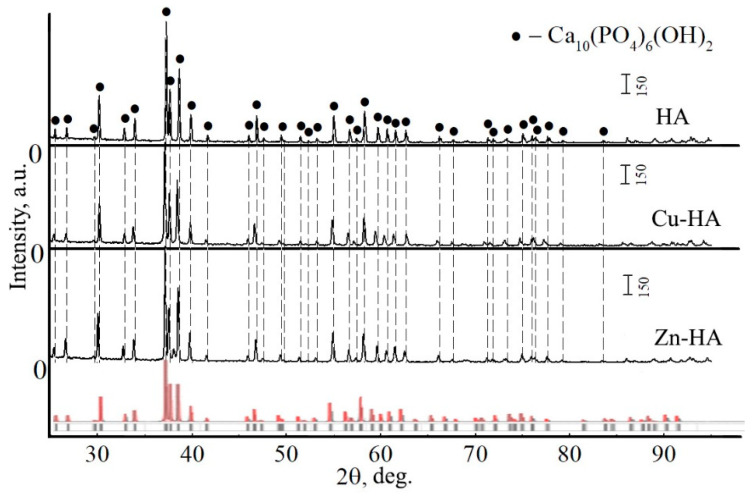
XRD patterns of HA, Cu-HA, and Zn-HA targets after sintering (red and gray lines represent HA etalon XRD-pattern according to PDF-card no. 00-009-0432).

Figure 4 shows the XRD-patterns of HA, Zn-HA, and Cu-HA targets. The composition of these targets is represented by a single phase, Ca_10_(PO_4_)_6_(OH)_2_. The main peaks of HA are observed. The calculated lattice parameters and unit cell volume for stoichiometric HA correspond to the reference values (Table 2).

It is worth noting the significant difference between the XRD-patterns obtained for the powder material and the targets after sintering. As can be seen, in the case of targets, the peaks are characterized by small broadenings and large intensities. This indicates a low level of residual stresses in the target material, low defectiveness, and large sizes of crystallites according to CSR sizes. This behavior may be due to the fact that during the target fabrication process, the powder material undergoes heat treatment, which leads to the relaxation of internal stresses and growth of the size of structural elements. XRD patterns of Zn- or Cu-HA targets as compared to stoichiometric HA have a small change. XRD patterns of Zn-HA and Cu-HA targets show a shift of HA phase reflexes toward smaller angles, relative to the XRD pattern of the stoichiometric HA target. These shifts indicate changes in lattice parameters and unit cell volume, which for Cu-HA were *a* = *b* = 9.4169 ± 0.0912 Å, c = 6.8796 ± 0.0153 Å, V = 528.4 Å^3^, and for Zn-HA were *a* = *b* = 9.4158 ± 0.0782 Å, *c* = 6.8911 ± 0.0096 Å, V = 529.1 Å^3^. The unit cell volume and lattice parameter *c* increased significantly during the synthesis of the target, indicating a significant rearrangement of the crystal structure. These changes are also in agreement with the previously published data for Zn-HA and Cu-HA powders after annealing at 1100 °C [18,43]. The results of the X-ray structural analysis of the targets are presented in Table 2.

In the work of Bhattacharjee A. et al. [51], it is noted that at an annealing temperature of 1100 °C the Zn-HA structure is transformed so that Zn^2+^ forms a linear O-Zn-O complex in the hexagonal OH channel (2b Wyckoff site). This transformation was reflected in an increase in the unit cell volume by 0.3%. A similar mechanism of O-Cu-O linear complex formation is observed for Cu-HA after annealing at 1150 °C, as described in [52]. Taking into account the fact that the synthesis of targets was performed at temperatures of 1000–1150 °C, and the unit cell volume of Cu-HA and Zn-HA significantly increased in comparison with the initial state, a similar mechanism of changing the position of Cu^2+^ or Zn^2+^ cations in the HA structure can be assumed. It should be noted that Cu-HA and Zn-HA targets are characterized by the highest values of X-ray density at close values of lattice parameters, which can contribute to the sputtering rate. The increase in apparent density of substituted HAs is also notable (Table 2).

**Figure 5:**
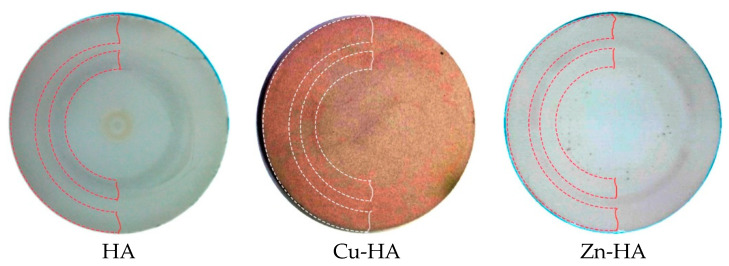
Digital photos of targets with a visible zone of erosion after multiple sputtering runs. Two areas of target erosion are highlighted in dotted lines for each photo.

Figure 5 shows photos of targets after several RF magnetron sputtering runs, which resulted in the work-time mean of 180 h per target. The images clearly show the zone of target erosion. It is also important to note that the target containing Cu^2+^ is brightly colored. The color change of the target confirms the incorporation of Cu^2+^ into the HA structure, as has already been shown by other researchers [53,54]. The incorporation of the Zn^2+^ cation into the HA structure affects the color change insignificantly.

**Figure 6:**
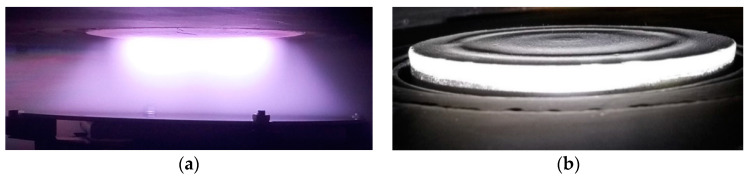
An RF magnetron discharge and a substrate holder (**a**), zone of HA target erosion after multiple deposition runs (**b**).

The arrangement of the target relative to the substrate holder during RF magnetron deposition is depicted in Figure 6a. From this image, the unbalanced nature of the magnetic system is obvious. Therefore, the fact that the coatings obtained in our study underwent ion and electron bombardment during their growth should be taken into account. It has already been reported that the use of unbalanced magnetron systems can lead to improved crystallinity or an increase in the adhesion of resulting coatings [55,56]. In Figure 6b a zone of HA target erosion is presented. As was depicted in Figure 5, erosion zone is comprised of two distinct areas that are highlighted in dotted lines and also visible in Figure 6b.

Due to the arrangement of the magnetic field in our magnetron setup, after multiple deposition runs a complex zone of erosion consisting of two tracks was visible which is represented in Figure 6b. Contrary to the more usual single erosion zone reported, for example in the work [57], in our case, the use of the target material as well as coating thickness homogeneity is significantly improved. However, the occurred zone of erosion does not significantly change the elemental composition of the deposited film.

In the end of this subsection, it could be concluded that the substitution of Ca^2+^ by Zn^2+^ or Cu^2+^ in the HA structure after mechanochemical synthesis does not lead to the formation of byproducts or secondary phases. When synthesizing targets from Cu-HA and Zn-HA powders, the lattice parameters and unit cell volume of crystalline HA increase significantly. The growth of unit cell volume during the synthesis of Cu-HA and Zn-HA targets can be connected with the change of Zn^2+^ or Cu^2+^ substituent ions position from Ca^2+^ ion position (II) in cationic sub-lattice to (OH)^−^ group substitution in apatite structure. All obtained targets are dense, pore-free, and suitable for the RF magnetron sputtering process.

### 3.2. Substituted HA Sputtering and Resulting ACP Coatings

**Figure 7:**
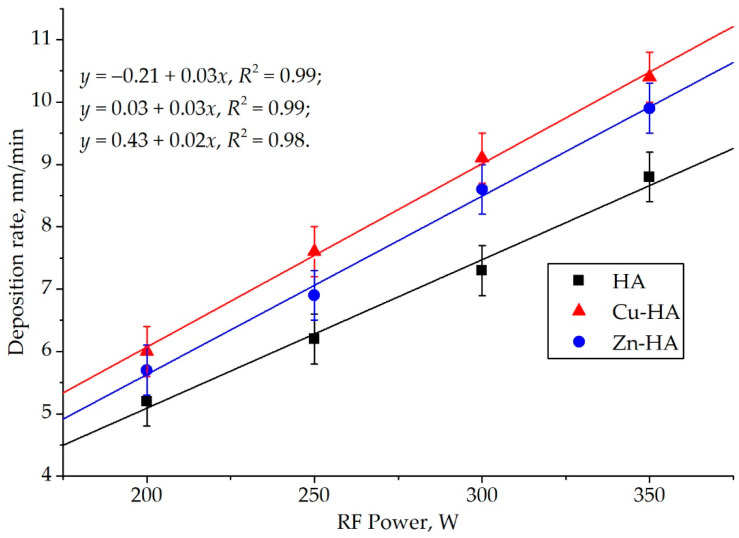
Coating growth rate obtained by sputtering of HA, Zn-HA, and Cu-HA targets vs. applied RF power.

To determine the most efficient mode of deposition of antibacterial and bioactive coatings containing Cu or Zn, a series of experiments were performed. In order to establish the influence of substituent ions in the HA structure on the deposition rate of CaP coatings, experiments were performed on sputtering targets at a fixed time and distance between the plane of the substrate holder and the target surface. The measurements were performed by ellipsometry on a monocrystalline Si wafer. The ellipsometry measurements also provided the information about the refractive index values *n* of the deposited coatings. The results are shown in Figure 7. It was found that the growth rate of CaP coatings increases linearly with increasing applied RF power. It was also found that HA targets having substituted ions in their structure are sputtered more efficiently, which is reflected in the increased growth rate of the coatings obtained from these targets relative to the HA target of stoichiometric composition. It is known that the sublimation rate is proportional to the density of the target material [58].

**Figure 8:**
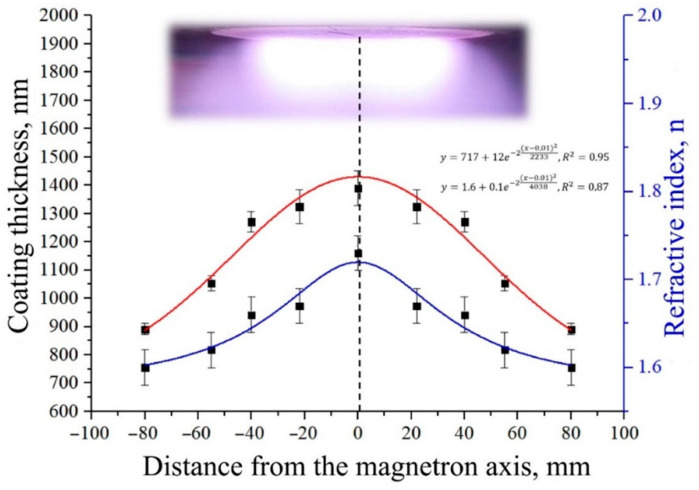
Thickness and refractive index distribution *n* of the CaP coating deposited from a Cu-HA target on the surface of the substrate holder.

As was shown earlier, the XRD and apparent density of the target material are significantly higher for Cu-HA and Zn-HA compared to stoichiometric HA. Therefore, it is assumed that in addition to the binding energy of atoms in substituted HA that is majorly governing the sputtering yield, a significant role is played by target density, which results in the modulation of the deposition rate. Figure 8 shows the thickness distribution of the RF magnetron coatings obtained during the sputtering of the Cu-HA target for three hours. The throw distance in this case was 70 mm.

From the presented distribution, we can conclude that the area of uniform deposition of CaP coatings is located in the diameter of 80 mm from the axis corresponding to the projection of the central axis of the target to the surface of the substrate holder. The size of the sputtering target (110 mm in diameter) allows to homogeneously deposit CaP coatings on multiple samples in one deposition run or functionalize the surface of dental implants. Moreover, the increased size of the target results in an increased area of RF discharge glow, which in turn allows working at lower Ar pressures. The refractive index *n* is considered to be an indicator of coating density because this parameter is linked to the material density and the presence of defects in its structure. The denser the packing of structural units, the greater the value of the refractive index according to [59,60]. Thus, we can conclude that the coating having the higher density value is growing at the center of the substrate holder. The coating density decreases slightly in the target erosion zone and further acquires a value lower than that for the value characteristic of stoichiometric HA (*n* = 1.64).

**Figure 9:**
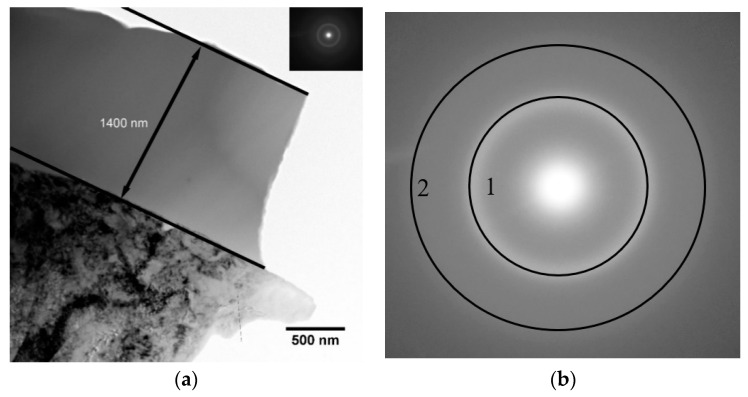

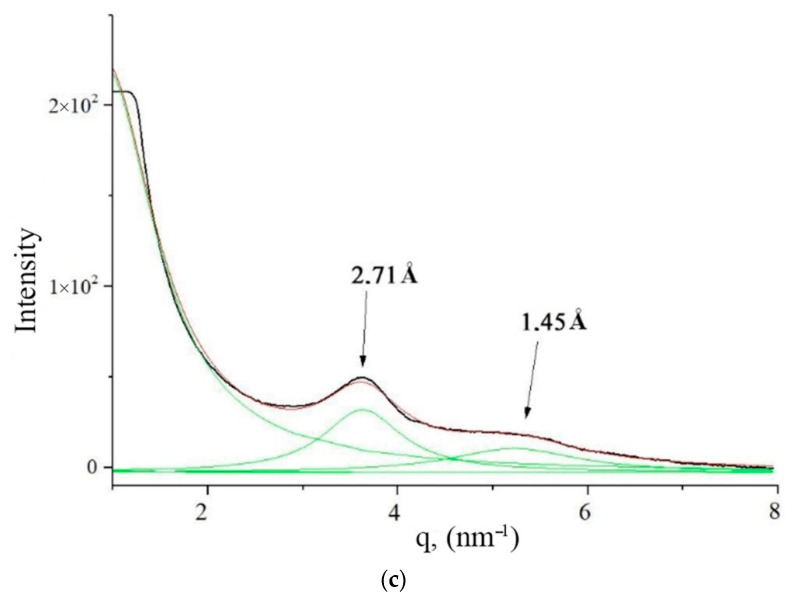
TEM image of the cross-section of the coating obtained by sputtering a Zn-HA target (**a**) with SAED (**b**) obtained from the coating area and the peaks of the two main diffusive halos, calculated from the SAED obtained from the amorphous layer (**c**).

In the case of Zn-HA and Cu-HA targets, it was found by TEM methods that the deposition on Ti substrates in the CG state leads to the formation of an amorphous, dense, homogeneous coating with the Ca/P ratio above stoichiometric and equal to 1.7–2.0 (Figure 9). The substrate temperature during the deposition process was recorded using a chromel-alumel thermocouple and did not exceed 100 °C. Figure 9a shows a cross-sectional image of the CaP coating on the Ti substrate. The bright-field TEM image clearly shows the interface between the coating and the substrate. No diffusion of coating elements into the substrate was observed. The coating is dense, without visible pores and defects. The selected area electron diffraction (SAED) obtained from the coating area is characterized by two diffusive halos with decreasing intensity, which is shown in Figure 9b. Figure 9c shows an averaged intensity of the diffracted electron wave, from which the peaks of the diffuse halos were determined. The center of the first diffusive halo is located at a distance of *d* = 2.71 Å, while the second ring corresponds to *d* = 1.45 Å. The calculated values correspond to the values of diffraction maxima from the {300}_HA_ and {304}_HA_ planes according to ICDD database. The appearance of the second halo was usually observed when imaging the thin regions of the sample, where the transmission thickness did not exceed 50 nm. When SAED is obtained from areas with a thickness of more than 50 nm, only the first, the brightest diffuse halo is observed. Thus, it can be concluded that under the conditions when the temperature of a CG Ti-substrate does not exceed 100 °C, and the coating is moderately bombarded by energy particles from RF magnetron discharge during growth, which is controlled by the distance from the target to the substrate plane, the ACP with the atomic near-order structure of crystalline HA is formed.

The Zn concentration that was analyzed using in-column EDX is low and is 0.4 ± 0.2 wt.% (0.3 ± 0.1 at.%). The concentration of Cu was found to be 0.5 ± 0.2 wt.% (0.4 ± 0.2 at.%). Hence, the concentration of Cu and Zn in the coatings does not exceed 0.5 wt.%, which is close to the content of these elements in the initial targets for sputtering coatings. It is reliably established that Cu and Zn are present in the respective coatings. However, the presented results of Cu and Zn element concentrations in the coatings cannot be considered quantitative (Table 3). Even though the content of Zn or Cu ions is small, an antibacterial effect has been detected in our previous study [61].

### 3.3. Formation of Calcium-Phosphate Coatings on a Titanium Substrate in the Nanostructured State

**Figure 10:**
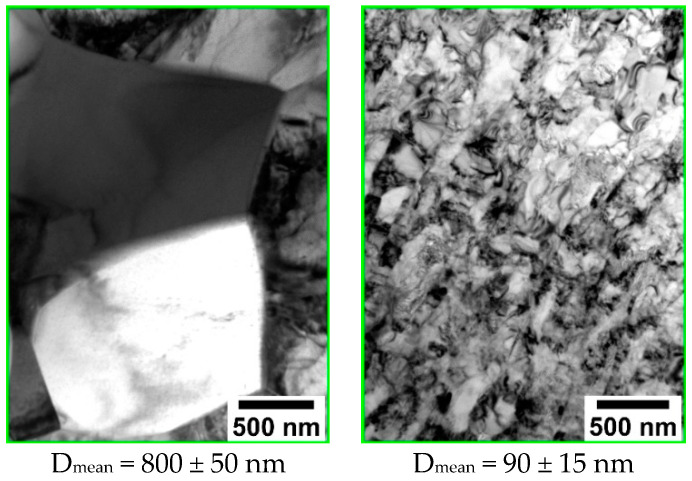
TEM images of the coarse grain (CG) and nanostructured (NS) Ti.

Since Ti-based alloys have already been extensively studied, it will be important to stress the difference in the microstructure of the materials used in the present work. The commercially pure Ti substrates in the CG state are represented by a coarse-crystalline structure, with an average grain size of 25–30 µm. The structure of the other substrates used in the work is presented in the TEM images (Figure 10). The grain size of the crystalline structure of the substrates varies from 90 to 800 nm. It is also worth noting that the stress state and dislocation density in the case of nanostructured (NS) Ti is much higher than in the case of substrates with a coarse-crystalline state. As it is known, the condensation of the coating during the PVD process is comprised of a random collision of atoms migrating over the surface; adhesion of adsorbed atoms on impurity or point defect or surface imperfections; and attachment to microrelief elements that play the role of crystallization centers. Thus, it is obvious that surface defects should significantly affect the condensation of coatings and their structural state. In this regard, it is important to study the deposition of CaP coatings on substrate with a different structural state.

**Figure 11:**
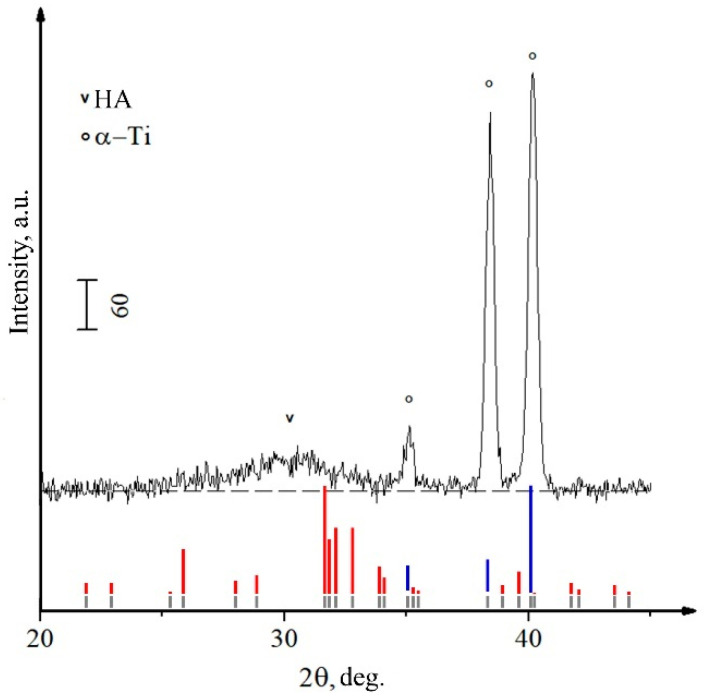
XRD pattern of the Zn containing CaP coating on the surface of NS Ti (red and gray lines represent Ti etalon XRD-patterns according to PDF-card no. 00-044-1294 HA etalon XRD-pattern according to PDF-card no. 00-009-0432).

In the case of coating deposition on substrates with significantly different structural states, a direct influence of surface defects, number of grain boundaries, and dislocation density on the growth and structure of the films is expected. Figure 11 shows an XRD-pattern of a coating deposited on the NS Ti surface after sputtering of a Zn-HA target, which was located at a distance of 40 mm from the plane of the substrate holder. The temperature of the substrate was 200 °C. A region of diffuse scattering was observed in the region corresponding to the range of angles in which the main reflexes of the HA crystal phase are located. According to the results of XRD, we can conclude that the coating is amorphous or the size of crystallites of the HA phase in the coating is so small that no individual reflexes of this phase can be detected by XRD.

**Figure 12:**
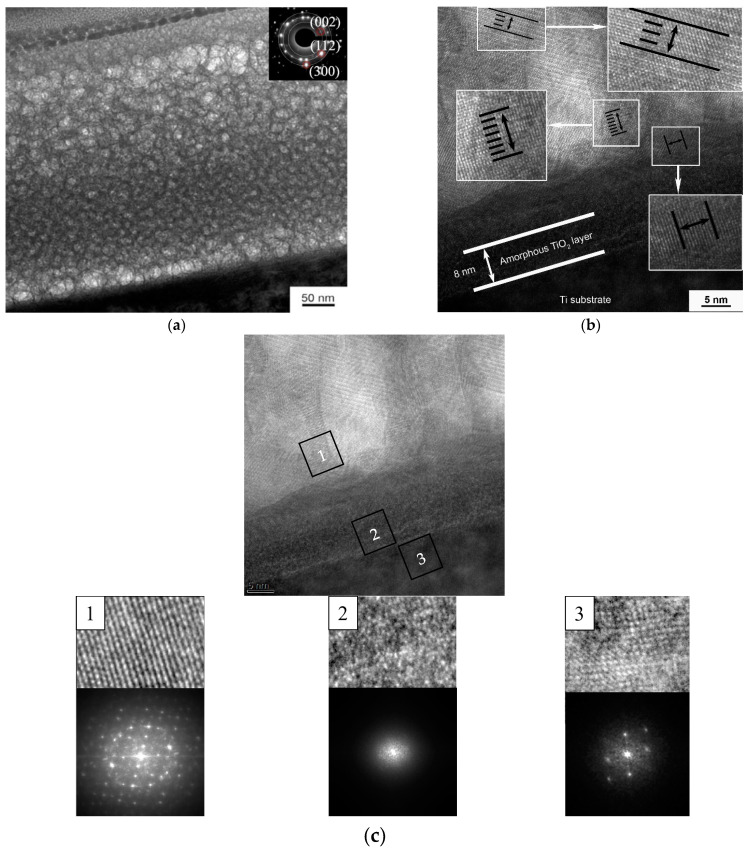
TEM image of the cross-section of Zn-HA coating on an NS Ti substrate with the corresponding SAED (**a**), high-resolution TEM image of the interface between the substrate and the coating (**b**), as well as the structure of coating areas (1), interface between the substrate and the coating (2) and substrate material (3) with the corresponding Fourier-transform image (**c**).

Figure 12 shows cross-sectional images of Zn-HA coatings on NS Ti substrate obtained by high-resolution TEM. The CaP coating is represented by an equiaxial polycrystalline grain structure with a gradient change in grain size (part of the grains are marked with circles in the figure as a visual guide) from the substrate to the coating surface in the range from 10 ± 3 nm to 27 ± 3 nm. This type of structure has the lowest internal residual stresses, which increases the mechanical properties of such coatings. The probability of cracking or chipping on the material surface with this type of structure is much lower compared to the coarse-grained structure. Figure 12b shows the coating-substrate interface in high resolution, from which the interplanar distances of crystallites included in the coating were calculated, which turned out to be 0.262 ± 0.006, 0.418 ± 0.007, 0.517 ± 0.007 nm, which corresponds to the reflection planes (300), (200), and (101) of the HA crystal lattice. The microdiffraction obtained from the coating area (Figure 12a) also indicates a polycrystalline type of structure with a fraction of the ACP phase. While the coating’s CaP structure is crystalline, the interface is amorphous TiO_2_, as evidenced by the dispersed salt-pepper type of contrast shown in Figure 12(c2), accompanied by characteristic scattering in the FFT image.

The Ca/P ratio was obtained by EDX mapping of the coating area and was 1.4. The Zn concentration was also small, as in the case of ACP, and did not exceed 0.3 at.%. This Zn concentration is close to the detection limit for the EDX method and can only indicate the presence of this element, but not its quantity in the material.

**Figure 13:**
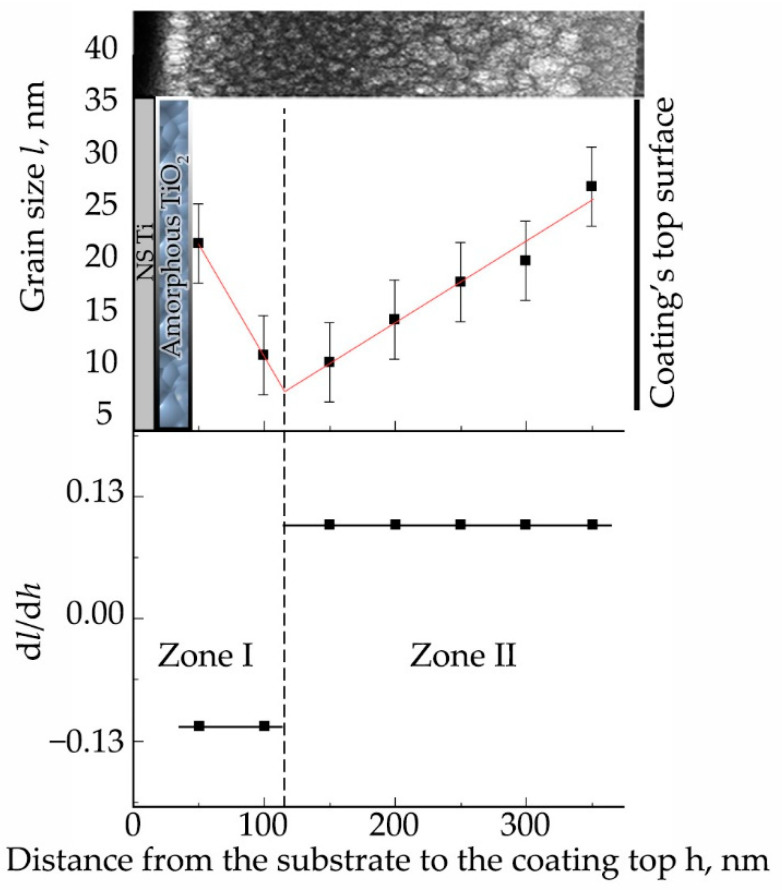
Grain size distribution in the coating (*l*) as a function of the distance (*h*) from the interface to the free surface of the coating in nm. The graph also shows the derivatives of the grain size distribution function d*l*/d*h*(*h*).

The equiaxed grain size distribution is a gradient of the grain size distribution function of the structure as a function of the distance between the substrate surface and the free coating surface. The grain size distribution function of the distance from the coating surface is shown in Figure 13. Thus, the grain size distribution can be divided into two zones. In zone I, at the initial stage of the coating growth the average grain size decreases, and the first CaP layers of the coating are formed. In zone II, there is a linear growth of the grain size, due to reduced heat dissipation, since the first deposited layers have a barrier function and prevent heat dissipation to the metallic NS Ti. The presented linear dependences associated with crystallite growth are reflected in the graph of the derivatives of the grain size distribution function d*l*/d*h*.

**Figure 14:**
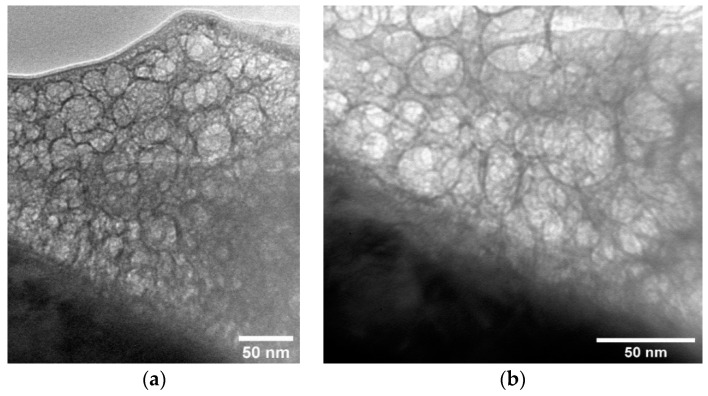
TEM image of Zn-HA coating cross-section on Ti substrate in the CG state (**a**) and the interface between the substrate and the coating (**b**).

However, the formation of a CaP coating on a CG Ti substrate also resulted in the formation of an equiaxed grain structure (Figure 14a), as in the case of NS Ti. The interface between the coating and the substrate is also clearly defined (Figure 14b). Thus, the determining parameter for the formation of one or another type of microstructure of the CaP coating is the temperature of the substrate rather than the structural state of the substrate material.

**Figure 15:**
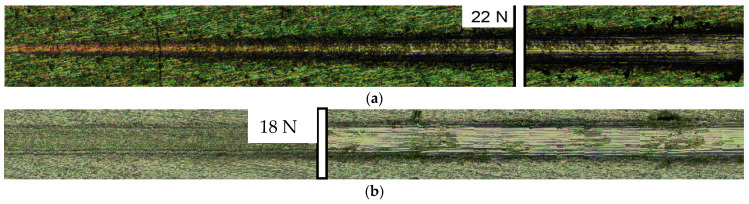
Indentation track from the indenter after the adhesion test by the scratch test of CaP coating on NS Ti substrate (**a**) and CG Ti substrate (**b**).

The transition layer plays a decisive role in the adhesion of the coating to the substrate surface. Thus, the amorphous TiO_2_ layer shown earlier in the TEM images helps to increase the adhesion of the coating to the substrate, such that the critical load in the scratch test was 22 N for the coating on NS Ti substrate and 18 N for the coating on CG Ti substrate (Figure 15). The mean value of critical load in the case of coatings deposited on NS Ti was 22 ± 2 N while for CG Ti it was found to be 18 ± 1 N.

On the other hand, the determined value of the critical load is probably related not only to the transition layer and the coating structure but also to the hardness of the NS Ti substrate. When the load on the indenter increases during the measurement, the failure of the coating is due to ductile fracture of the substrate, which is not observed in the case of NS Ti substrate. Hence, NS Ti is a favorable choice when increased mechanical properties of the implant are needed.

### 3.4. Influence of Controlled Heating of Substrates during RF Magnetron Sputtering and Post-Deposition Heat Treatment

**Figure 16:**
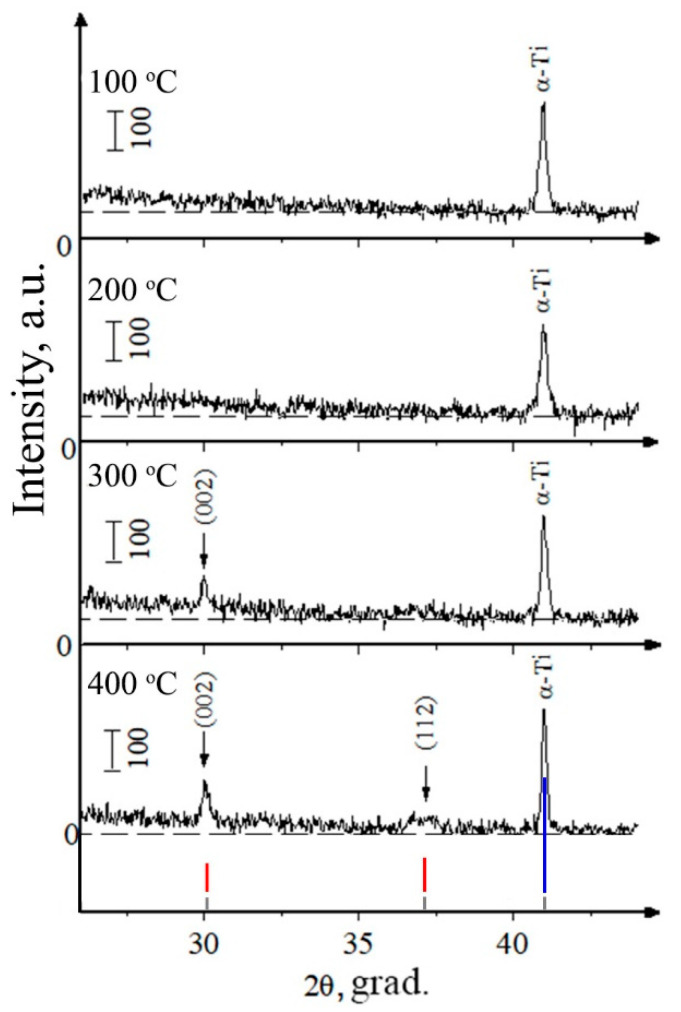
XRD of CaP coating formed at substrate temperatures of 100, 200, 300, and 400 °C. Crystallographic planes are marked for the HA phase (red, blue, and gray lines represent HA (red) and Ti (blue) etalon XRD patterns according to PDF-card no. 00-009-0432 and 00-044-1294, respectively).

As was mentioned in the previous subsection, the main governing parameter of coatings’ structure formation is temperature. In this subsection, the influence of controlled heating of substrates during RF magnetron sputtering and post-deposition HT are going to be discussed. The XRD of samples deposited at different substrate temperatures using the heater mounted in the substrate holder shows that the initial temperature for the formation of coating with the formation of texture and growth of crystallites with predominant orientation in the direction [002] is 300 °C (Figure 16). Increasing the temperature to 400 °C leads to an increase in the degree of crystallinity and to the appearance of the reflections from (112) plane. Predominant growth of crystallites in the plane (002) is associated with the fact that this direction is the most energetically advantageous, has the lowest surface energy, and is characteristic of HA-based coatings. The influence of Zn or Cu ions did not result in a significant change in the coating structure. It is connected with a small concentration of dopant material in the coating.

**Figure 17:**
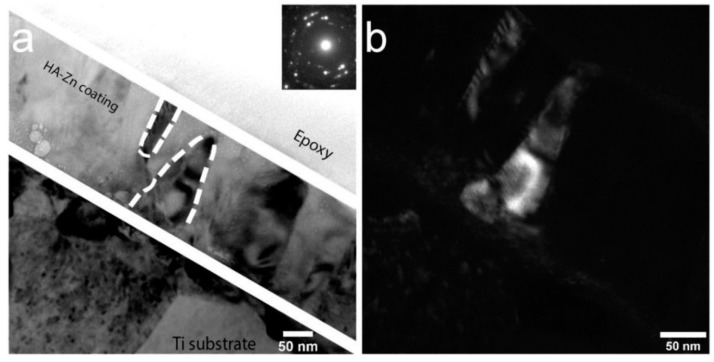
TEM image of the cross-section of Zn-HA coating on Ti deposited at the substrate temperature of 400 °C bright field, the inset shows the corresponding SAED (**a**) and dark field (**b**) [62].

The structure of the Zn-HA coating has been recently published [62]. Figure 17 shows TEM images of polycrystalline Zn-HA coating deposited on a substrate controllably heated up to 400 °C. Using the microdiffraction pattern obtained from the coating (Figure 17a), it was possible to determine the crystallographic planes of growth—(002), (102), (211), and (112). As in the previous case, a thin TiO_2_ oxide layer is visible at the interface between the coating and the substrate. It is worth noting that the deposition of CaP coatings on a hot substrate significantly slows down the coating growth rate, which in this case was 0.9 nm/min. Figure 17b shows a dark-field image of the Zn-HA coating in the reflection (002). A fine zoned structure is represented by densely packed HA grains and subgrains. The columnar type of coating growth is visible, the average grain size, calculated from several sections, is 30–90 nm. The formed coating is sub-stoichiometric with a Ca/P ratio equal to 1.5, which is associated with active desorption of phosphate groups under the influence of high substrate temperature during deposition. The Zn content in the coating does not exceed 0.3 at.%.

**Figure 18:**
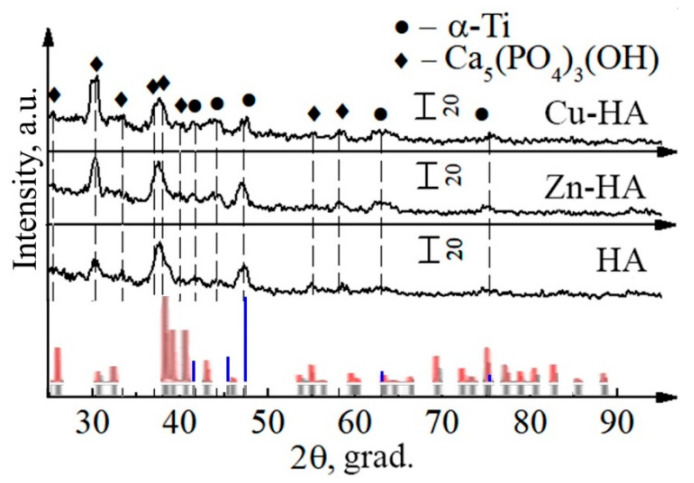
XRD of the CaP coatings after post-deposition annealing in air (red, blue, and gray lines represent HA (red) and Ti (blue) etalon XRD patterns according to PDF-card no. 00-009-0432 and 00-044-1294, respectively).

In order to keep a high deposition rate and obtain crystalline HA structure, it is far more convenient to perform a post-deposition HT. For crystallization of the coatings deposited from HA, Zn-HA, and Cu-HA targets, annealing in an air atmosphere was performed. The Ti samples were annealed for 1 h at a constant temperature of 700 °C. Figure 18 shows the XRD of the coatings obtained after sputtering of Zn- or Cu-HA targets and stoichiometric HA after annealing. It can be seen that the XRD patterns obtained from these coatings are represented by a single main phase—HA. The performed annealing made it possible to crystallize the coating so that the main reflexes characteristic of the HA phase are clearly defined.

Table 4 shows the lattice parameters of HA coatings on Ti substrates after annealing. It is found that the calculated lattice parameters agree with the theoretical values for pure HA. The substitution of Zn^2+^ and Cu^2+^ cations in the HA crystal lattice is confirmed both by an increase in the lattice volume from 3 to 4% compared to stoichiometric HA and with a regular increase in the unit cell parameter *c*, as has been demonstrated earlier for Cu-HA or Zn-HA targets. Thus, the coatings obtained by RF magnetron sputtering of targets sintered from pure HA, Zn-HA, and Cu-HA powders on Ti substrates are characterized by an amorphous structure. Annealing in the furnace in air with a stepwise temperature increase allows the transformation of the CaP coatings to the crystalline state close to the stoichiometric HA phase.

**Figure 19:**
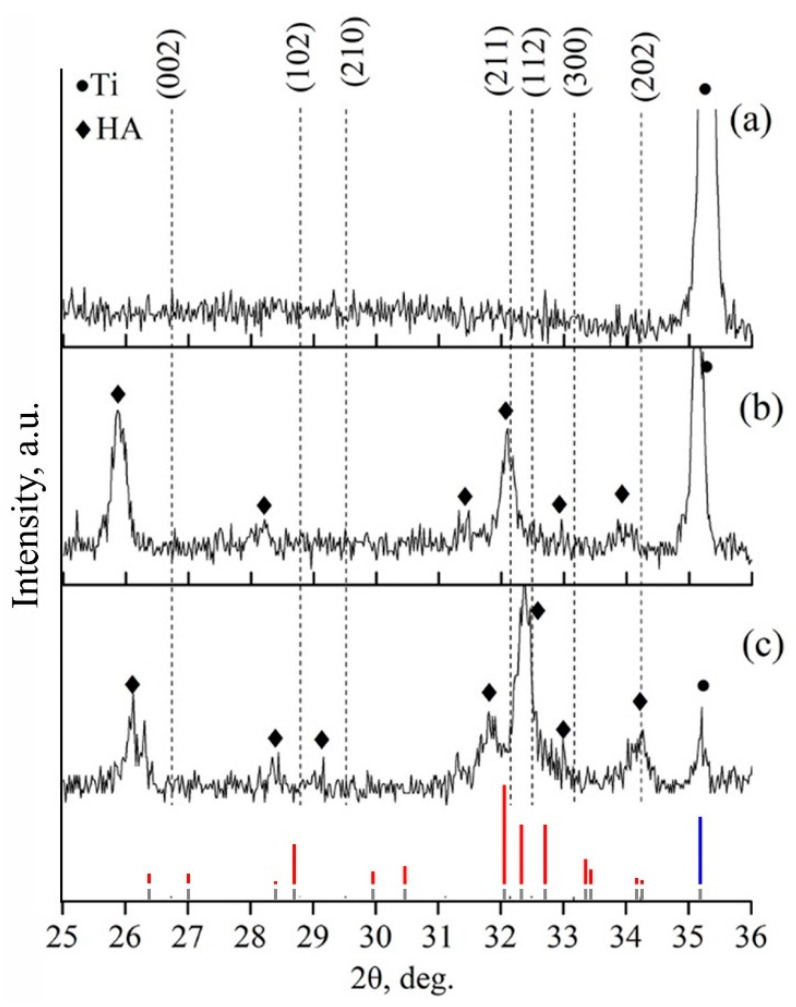
XRD-patterns of Cu-HA coating on Ti as-deposited (**a**), after annealing at 400 °C for 3 h (**b**), and after HT at 700 °C for 3 h (**c**) (red, blue, and gray lines represent HA (red) and Ti (blue) etalon XRD patterns according to PDF-card no. 00-009-0432 and 00-044-1294, respectively).

To compare the influence of an ambient atmosphere during annealing on the crystallinity state of coatings, the next annealing processes were performed in an Ar gas atmosphere. The XRD results are shown in Figure 19. From the results, it can be seen that at 400 °C a crystal structure characteristic of the HA lattice is formed with the preferential growth direction, which corresponds to the crystallographic direction <001> corresponding to (002) plane.

The broadening of the reflexes identified on the XRD-patterns relative to the reflexes characteristic of stoichiometric HA indicates the development of internal stresses in the coating. At the maximum annealing temperature of 700 °C, the value of the CSR size is smaller than at 400 °C which could be connected to the faster grain growth and more competing character of grain evolution (Table 5). From Figure 19a, it is visible that the as-deposited coating is in an amorphous state. Coating post-deposition HT at 400 °C in a protective Ar atmosphere leads to the rearrangement of Ca (Cu, Zn), P, O, H atoms and the formation of a texturized HA-lattice (Figure 19b). As can be seen in Figure 19b, the highest intensity belongs to the (002) peak, i.e., <001> is the direction of preferable growth for the PVD coatings in general. At the initial moment of coating crystallization, the crystallites grow in the direction according to the temperature gradient, i.e., in an orientation perpendicular to the substrate surface. After that, at a higher HT temperature of 700 °C, as can be seen from Figure 19c, orientation planes (211) or (112) start to appear. Structure rearrangement resulted in the XRD pattern being more closely related to the stochiometric HA. In contrast to that, in the case of post-deposition HT at 700 °C in air, a texturized structure following the growth direction of <001> appears similar to what was observed for HT at a lower temperature of 400 °C in the Ar atmosphere. It could be so that coating crystallization in air or Ar atmosphere is drastically different. Crystallization in the Ar atmosphere could start from lower treatment temperatures while the higher temperature is required for similar to stochiometric HA lattice formation in air. We aim to perform additional experiments and thoroughly discuss the mechanism of CaP coating crystallization in different atmosphere conditions.

There are certain limitations to the current study that need to be mentioned. To precisely determine the position of substituents in the HA lattice and more precisely analyze an amorphous CaP coating, more advanced techniques are needed, for example, synchrotron X-ray studies, Raman spectroscopy of FTIR spectroscopy, small-angle X-ray scattering, or nuclear magnetic resonance studies. Frequently, to determine the elemental composition of the obtained powders, sintered targets, and deposited coatings, an EDX method is routinely used; however, the quantitative results are somewhat difficult to obtain due to rather small concentrations of substitution elements. For further studies, more advanced techniques, such as X-ray photoelectron spectroscopy, are suggested. Nevertheless, the concentration of Zn or Cu provided the antibacterial effect in vitro that was shown in our previous report [61]; it is worth increasing the concentration of these dopants to determine an upper and lower limit of antibacterial effect that does not result in systemic cytotoxicity. Moreover, it is necessary to conduct experiments regarding the influence of coatings’ crystallinity states on their degradation behavior and antibacterial properties both in vitro and in vivo.

## 4. Conclusions

It was shown in the present paper how the elemental composition and structure of targets, substrates grain distribution, and thermal treatments could be used to fine-tune the deposited antibacterial coatings. It was found that substitutions of Cu^+2^ or Zn^+2^ cations in the lattice of hydroxyapatite increase the growth rate of coatings. It was established that the deposition of coatings by RF magnetron sputtering on titanium substrates both in the coarse-crystalline and nanostructured state with the temperature of 200 °C leads to the formation of an equiaxed nanograined structure with a gradient in its size. The use of nanostructured titanium, however, notably increased the scratch resistance. It was shown that the substrate temperature regulated by controlled substrate heating in the range (100–400) °C determines the crystal structure of the coating, from amorphous (<100 °C) to nanocrystalline (200 °C) and further to columnar (>300 °C). At a substrate temperature of 400 °C, a coating with a columnar grain structure is formed with the predominant grain direction perpendicular to the substrate plane and oriented in the [002]_HA_ direction. The range of transverse size of the “columnar” grains is 30–90 nm. It was revealed that post-deposition heat treatment in an air atmosphere and an argon atmosphere in the temperature range of 400–700 °C allows the transformation of amorphous calcium phosphate coatings into a crystalline state without damaging their integrity. Further research will be devoted to the investigation of Cu and Zn containing calcium phosphates at higher concentrations and the influence of coatings’ crystallinity states on their degradation behavior and antibacterial properties both in vitro and in vivo.

## Figures and Tables

**Table 1:** Results of X-ray diffraction analysis of powders of HA, Cu-HA, and Zn-HA.

Powder	*a = b*, Å	*c*, Å	V, Å^3^	CSR, nm
HA	9.4166 ± 0.0021	6.8775 ± 0.005	528.1	94 ± 21
Cu-HA	9.4201 ± 0.0611	6.7659 ± 0.2546	519.9	74 ± 13
Zn-HA	9.4311 ± 0.0074	6.6649 ± 0.425	513.4	76 ± 29
ICDD	9.422	6.881	533.7	

**Table 2:** Results of X-ray diffraction analysis and density measurements of HA, Cu-HA, and Zn-HA targets.

Target	*a = b*, Å	*c*, Å	V, Å^3^	$\rho_{relative}$, g/cm^3^	$\rho_{XRD}$, g/cm^3^
HA	9.4175 ± 0.0035	6.8767 ± 0.0082	528.1	2.64 ± 0.12	3.158
Cu-HA	9.4169 ± 0.0912	6.8796 ± 0.0153	519.9	2.8 ± 0.08	3.172
Zn-HA	9.4158 ± 0.0782	6.8911 ± 0.0096	513.4	2.83 ± 0.14	3.185
ICDD	9.422	6.881	533.7	-	3.154

**Table 3:** Ca/P ratio and Zn and Cu content in CaP coatings.

Target Material	Ca/P	Zn, wt.%	Cu, wt.%
HA	1.9	-	-
Cu-HA	2.5	-	0.3
Zn-HA	2.25	0.8	-

**Table 4:** Lattice parameters and crystallinity index of CaP coatings deposited from different targets.

Target Material	Lattice Parameters	Crystallinity Index (%)
HA	*a* = *b* = 9.529 Å, *c* = 6.841 Å	18 ± 12
Cu-HA	*a* = *b* = 9.659 Å, *c* = 6.869 Å	25 ± 17
Zn-HA	*a* = *b* = 9.587 Å, *c* = 6.913 Å	29 ± 14

**Table 5:** Crystalline lattice parameters in the structure of Cu-HA coatings obtained by sputtering Cu-HA target on Ti substrates after annealing at 400 or 700 °C, 3 h.

Annealing Temperature, °C	Lattice Parameters	CSR, nm	Crystallinity Index (%)
-	Amorphous state	-	-
400	*a* = *b* = 9.547Å, *c* = 6.866 Å	47	27 ± 12
700	*a* = *b* = 9.534 Å, *c* = 6.783 Å	13	43 ± 18

## Data Availability

Data is contained within the article.
